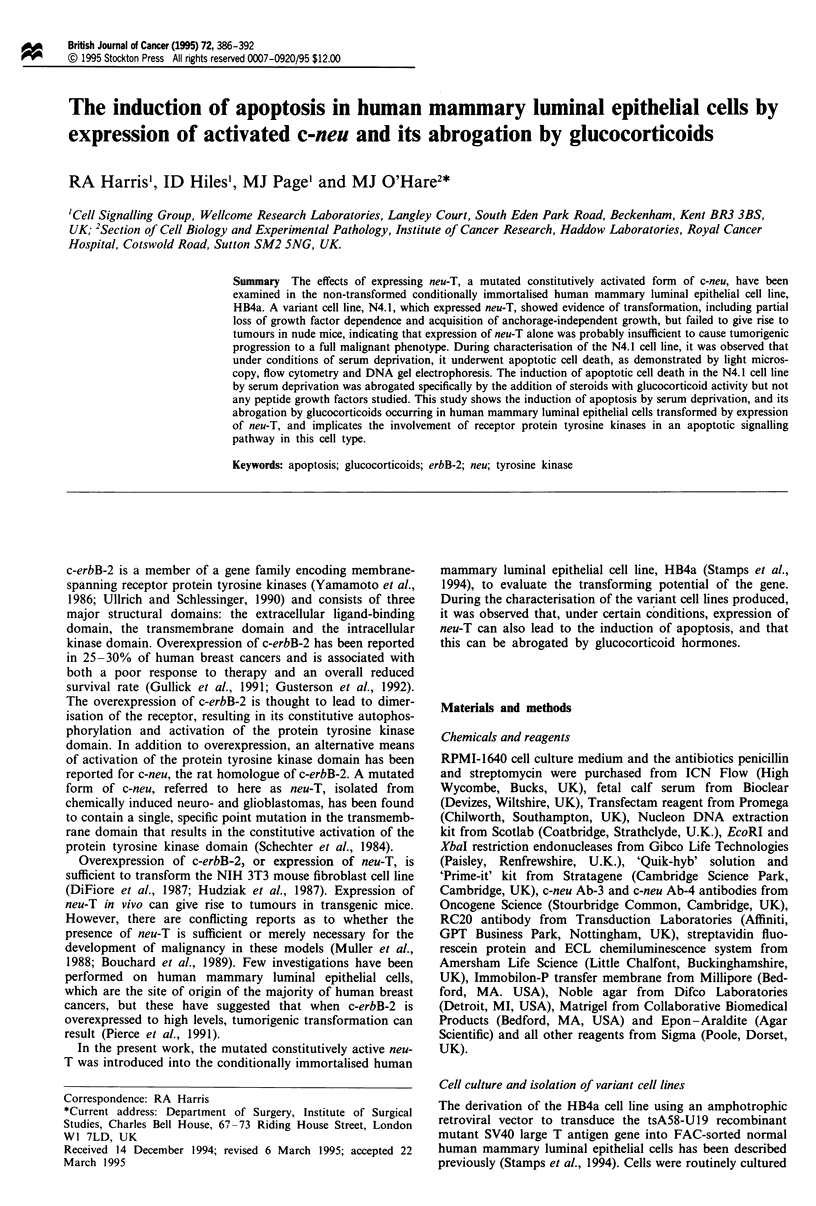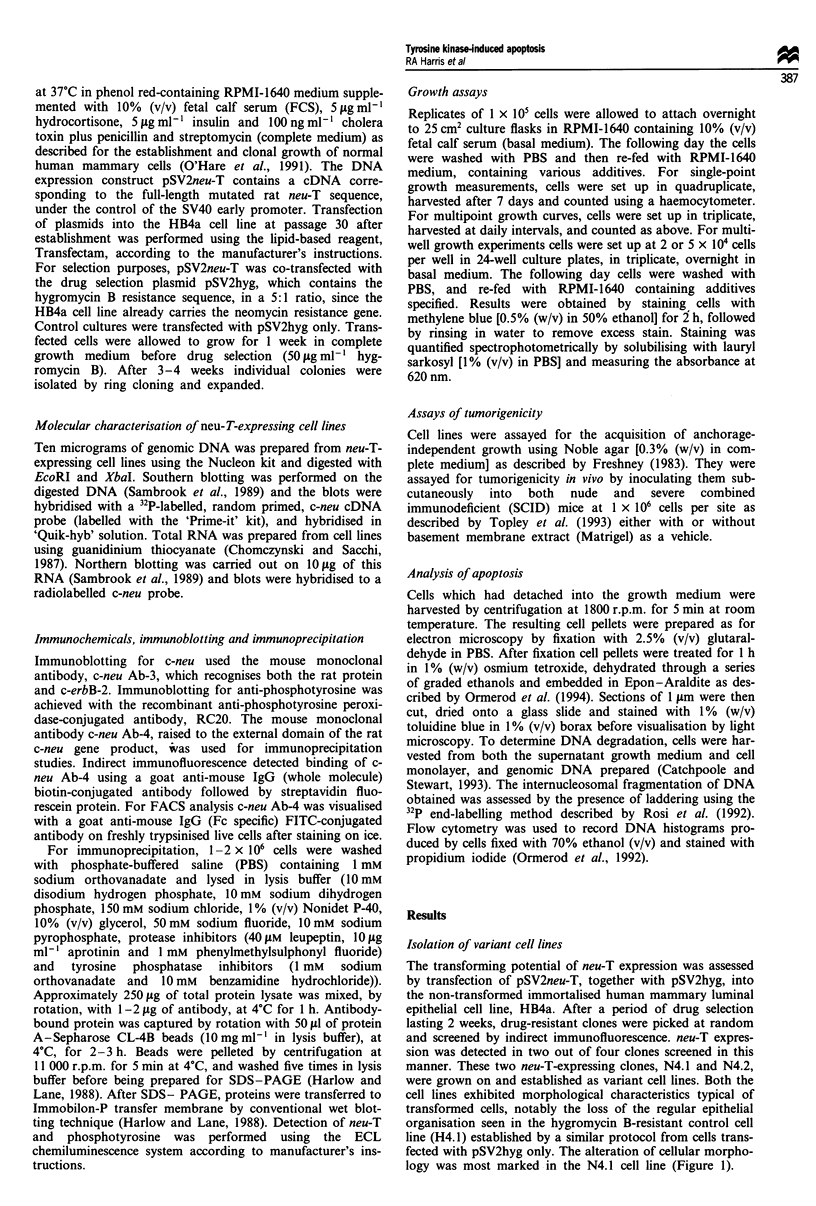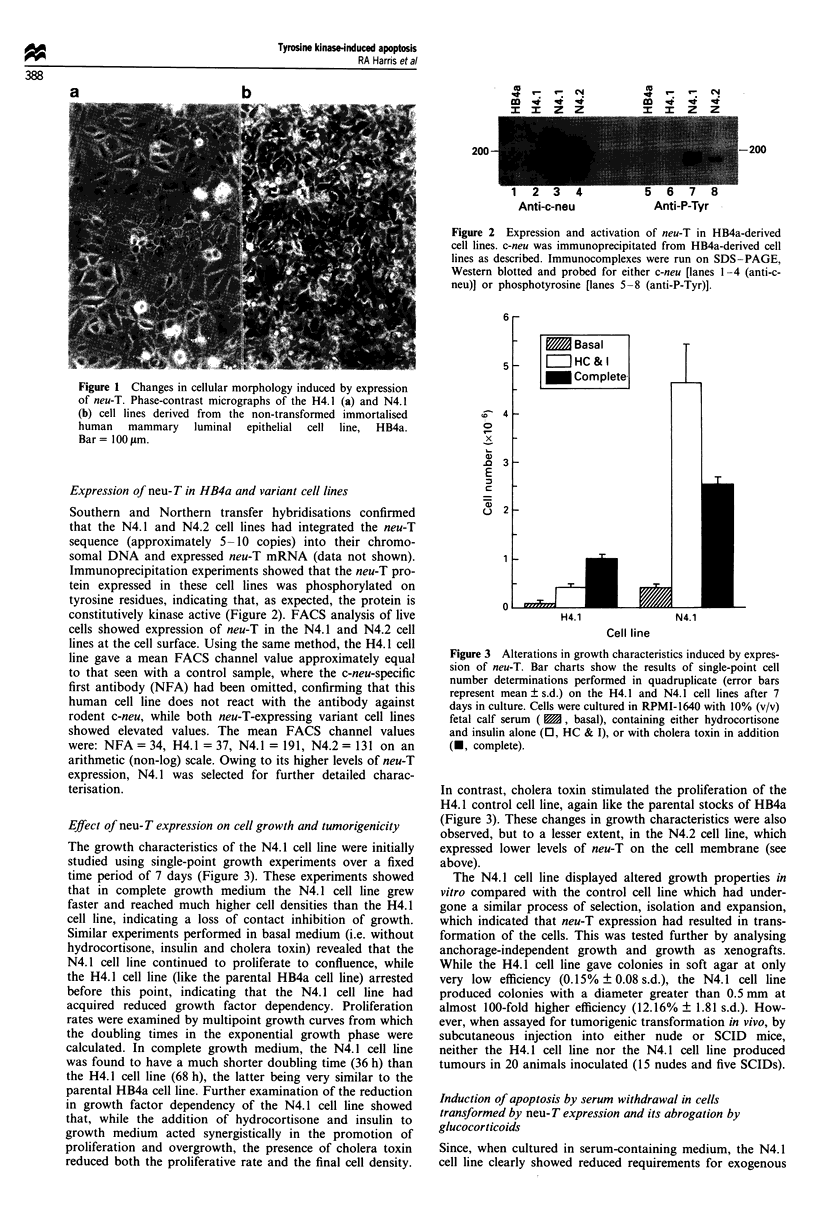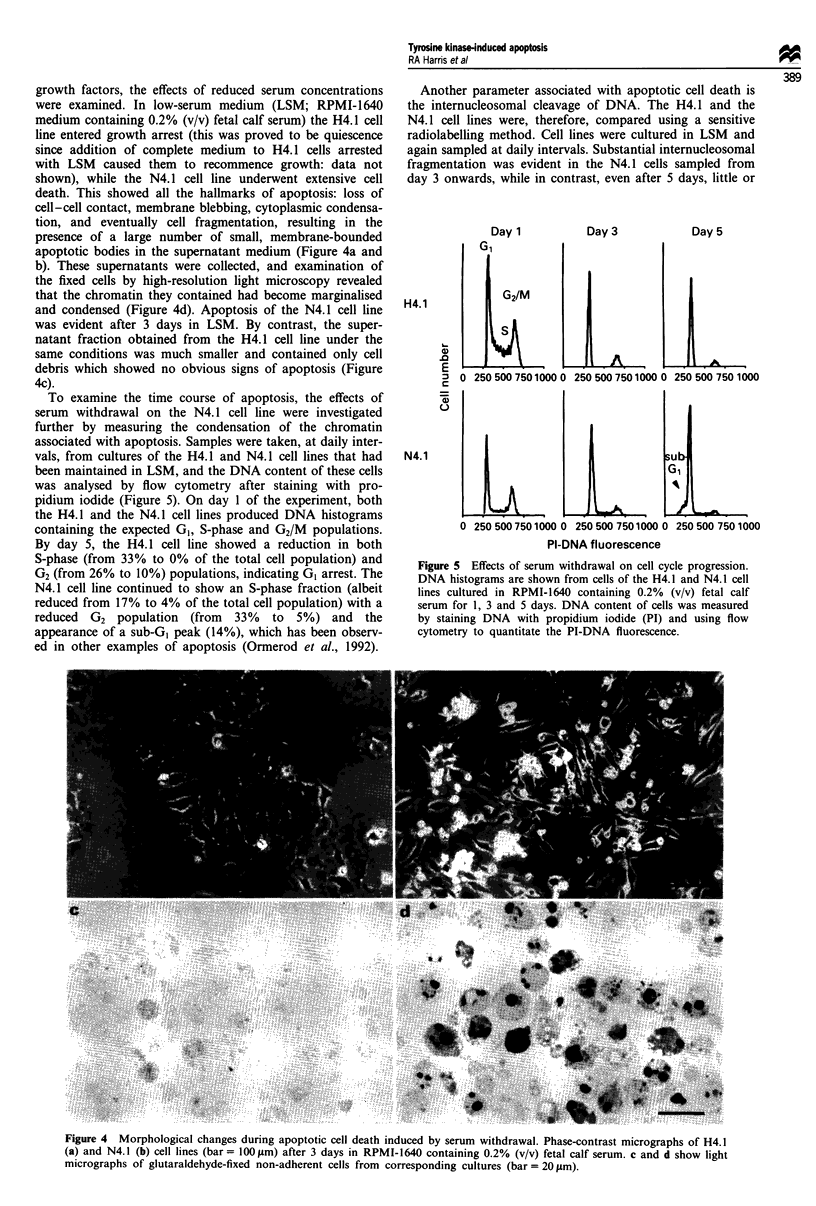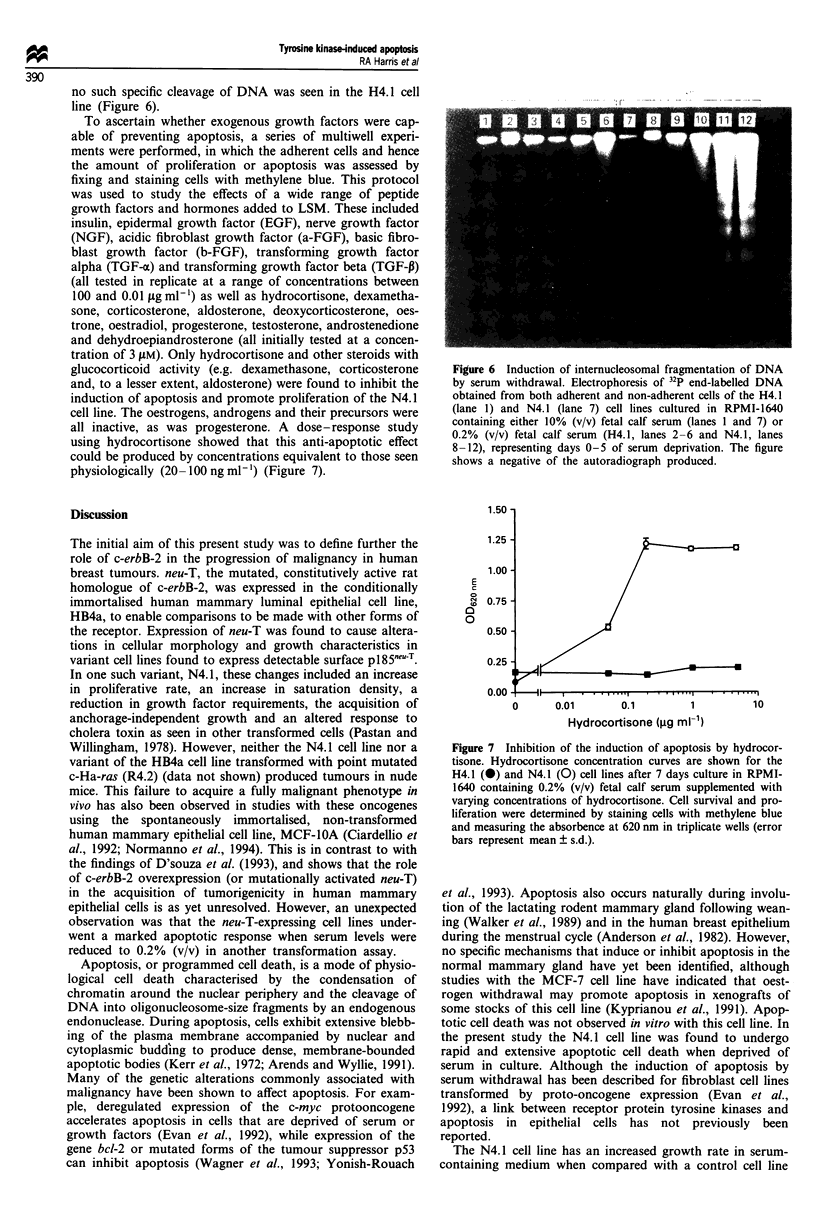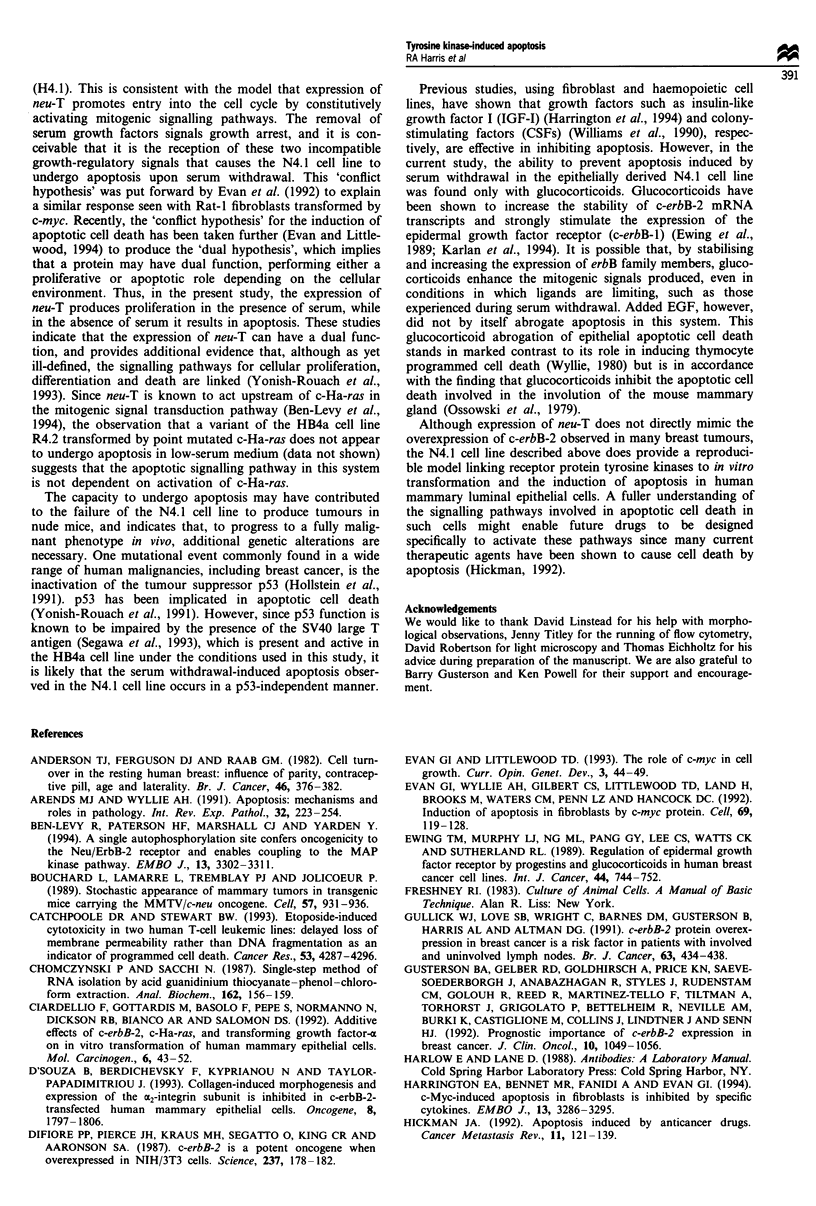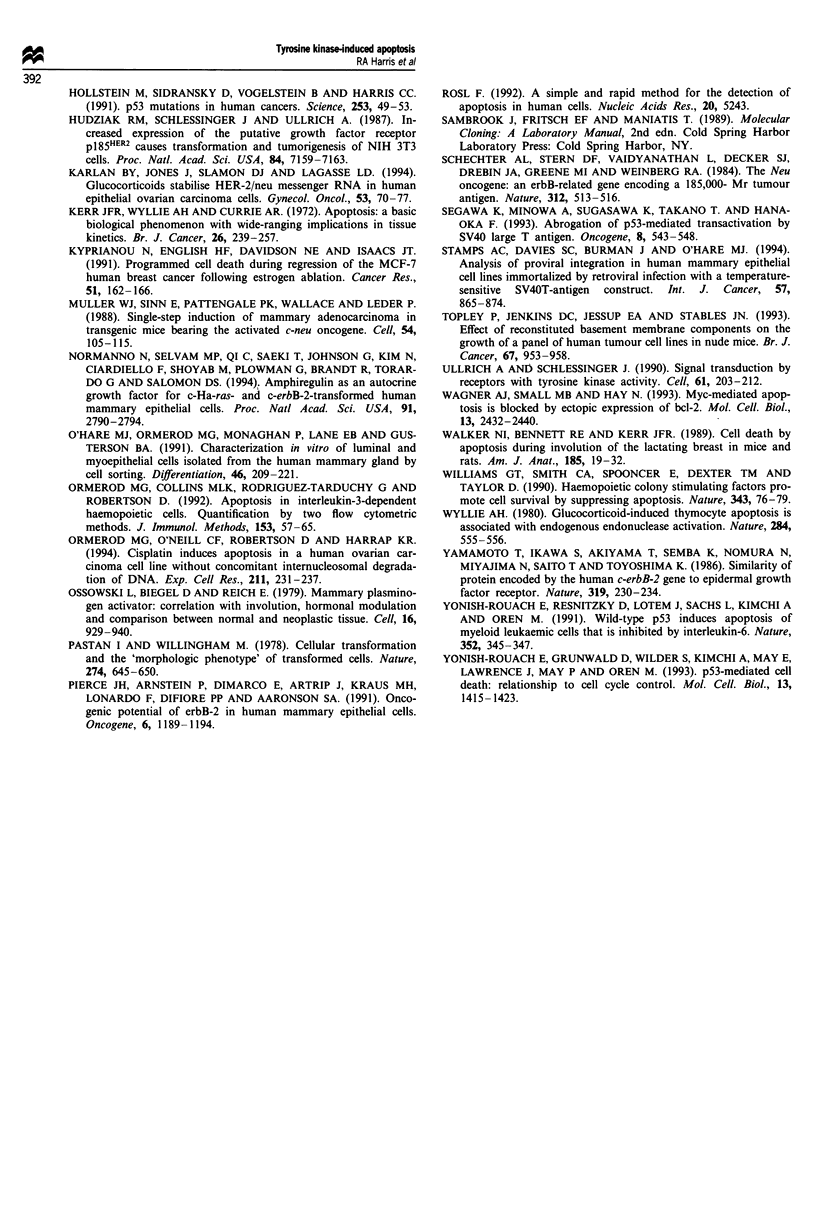# The induction of apoptosis in human mammary luminal epithelial cells by expression of activated c-neu and its abrogation by glucocorticoids.

**DOI:** 10.1038/bjc.1995.343

**Published:** 1995-08

**Authors:** R. A. Harris, I. D. Hiles, M. J. Page, M. J. O'Hare

**Affiliations:** Cell Signalling Group, Wellcome Research Laboratories, Beckenham, Kent, UK.

## Abstract

**Images:**


British Journal of Cancer (1995) 72, 386-392
© 1995 Stockton Press All rights reserved 0007-0920/95 $12.00

### The induction of apoptosis in human mammary luminal epithelial cells by expression of activated c-*neu* and its abrogation by glucocorticoids

RA Harris[1], ID Hiles[1], MJ Page[1] and MJ O'Hare[2]*

[1]*Cell Signalling Group, Wellcome Research Laboratories, Langley Court, South Eden Park Road, Beckenham, Kent BR3 3BS, UK;* [2]*Section of Cell Biology and Experimental Pathology, Institute of Cancer Research, Haddow Laboratories, Royal Cancer Hospital, Cotswold Road, Sutton SM2 5NG, UK.*

**Summary** The effects of expressing *neu*-T, a mutated constitutively activated form of c-*neu*, have been examined in the non-transformed conditionally immortalised human mammary luminal epithelial cell line, HB4a. A variant cell line, N4.1, which expressed *neu*-T, showed evidence of transformation, including partial loss of growth factor dependence and acquisition of anchorage-independent growth, but failed to give rise to tumours in nude mice, indicating that expression of *neu*-T alone was probably insufficient to cause tumorigenic progression to a full malignant phenotype. During characterisation of the N4.1 cell line, it was observed that under conditions of serum deprivation, it underwent apoptotic cell death, as demonstrated by light microscopy, flow cytometry and DNA gel electrophoresis. The induction of apoptotic cell death in the N4.1 cell line by serum deprivation was abrogated specifically by the addition of steroids with glucocorticoid activity but not any peptide growth factors studied. This study shows the induction of apoptosis by serum deprivation, and its abrogation by glucocorticoids occurring in human mammary luminal epithelial cells transformed by expression of *neu*-T, and implicates the involvement of receptor protein tyrosine kinases in an apoptotic signalling pathway in this cell type.

**Keywords:** apoptosis; glucocorticoids; *erb*B-2; *neu*; tyrosine kinase c-*erb*B-2 is a member of a gene family encoding membrane-spanning receptor protein tyrosine kinases (Yamamoto *et al.*, 1986; Ullrich and Schlessinger, 1990) and consists of three major structural domains: the extracellular ligand-binding domain, the transmembrane domain and the intracellular kinase domain. Overexpression of c-*erb*B-2 has been reported in 25–30% of human breast cancers and is associated with both a poor response to therapy and an overall reduced survival rate (Gullick *et al.*, 1991; Gusterson *et al.*, 1992). The overexpression of c-*erb*B-2 is thought to lead to dimerisation of the receptor, resulting in its constitutive autophosphorylation and activation of the protein tyrosine kinase domain. In addition to overexpression, an alternative means of activation of the protein tyrosine kinase domain has been reported for c-*neu*, the rat homologue of c-*erb*B-2. A mutated form of c-*neu*, referred to here as *neu*-T, isolated from chemically induced neuro- and glioblastomas, has been found to contain a single, specific point mutation in the transmembrane domain that results in the constitutive activation of the protein tyrosine kinase domain (Schechter *et al.*, 1984).

Overexpression of c-*erb*B-2, or expression of *neu*-T, is sufficient to transform the NIH 3T3 mouse fibroblast cell line (DiFiore *et al.*, 1987; Hudziak *et al.*, 1987). Expression of *neu*-T *in vivo* can give rise to tumours in transgenic mice. However, there are conflicting reports as to whether the presence of *neu*-T is sufficient or merely necessary for the development of malignancy in these models (Muller *et al.*, 1988; Bouchard *et al.*, 1989). Few investigations have been performed on human mammary luminal epithelial cells, which are the site of origin of the majority of human breast cancers, but these have suggested that when c-*erb*B-2 is overexpressed to high levels, tumorigenic transformation can result (Pierce *et al.*, 1991).

In the present work, the mutated constitutively active *neu*-T was introduced into the conditionally immortalised human mammary luminal epithelial cell line, HB4a (Stamps *et al.*, 1994), to evaluate the transforming potential of the gene. During the characterisation of the variant cell lines produced, it was observed that, under certain conditions, expression of *neu*-T can also lead to the induction of apoptosis, and that this can be abrogated by glucocorticoid hormones.

Correspondence: RA Harris
*Current address: Department of Surgery, Institute of Surgical Studies, Charles Bell House, 67–73 Riding House Street, London W1 7LD, UK
Received 14 December 1994; revised 6 March 1995; accepted 22 March 1995

### Materials and methods

### *Chemicals and reagents*

RPMI-1640 cell culture medium and the antibiotics penicillin and streptomycin were purchased from ICN Flow (High Wycombe, Bucks, UK), fetal calf serum from Bioclear (Devizes, Wiltshire, UK), Transfectam reagent from Promega (Chilworth, Southampton, UK), Nucleon DNA extraction kit from Scotlab (Coatbridge, Strathclyde, U.K.), *Eco*RI and *Xba*I restriction endonucleases from Gibco Life Technologies (Paisley, Renfrewshire, U.K.), 'Quik-hyb' solution and 'Prime-it' kit from Stratagene (Cambridge Science Park, Cambridge, UK), c-*neu* Ab-3 and c-*neu* Ab-4 antibodies from Oncogene Science (Stourbridge Common, Cambridge, UK), RC20 antibody from Transduction Laboratories (Affiniti, GPT Business Park, Nottingham, UK), streptavidin fluorescein protein and ECL chemiluminescence system from Amersham Life Science (Little Chalfont, Buckinghamshire, UK), Immobilon-P transfer membrane from Millipore (Bedford, MA. USA), Noble agar from Difco Laboratories (Detroit, MI, USA), Matrigel from Collaborative Biomedical Products (Bedford, MA, USA) and Epon–Araldite (Agar Scientific) and all other reagents from Sigma (Poole, Dorset, UK).

### *Cell culture and isolation of variant cell lines*

The derivation of the HB4a cell line using an amphotrophic retroviral vector to transduce the tsA58-U19 recombinant mutant SV40 large T antigen gene into FAC-sorted normal human mammary luminal epithelial cells has been described previously (Stamps *et al.*, 1994). Cells were routinely cultured at 37°C in phenol red-containing RPMI-1640 medium supplemented with 10% (v/v) fetal calf serum (FCS), 5 $\mu$g ml$^{-1}$ hydrocortisone, 5 $\mu$g ml$^{-1}$ insulin and 100 ng ml$^{-1}$ cholera toxin plus penicillin and streptomycin (complete medium) as described for the establishment and clonal growth of normal human mammary cells (O'Hare *et al.*, 1991). The DNA expression construct pSV2*neu*-T contains a cDNA corresponding to the full-length mutated rat *neu*-T sequence, under the control of the SV40 early promoter. Transfection of plasmids into the HB4a cell line at passage 30 after establishment was performed using the lipid-based reagent, Transfectam, according to the manufacturer's instructions. For selection purposes, pSV2*neu*-T was co-transfected with the drug selection plasmid pSV2hyg, which contains the hygromycin B resistance sequence, in a 5:1 ratio, since the HB4a cell line already carries the neomycin resistance gene. Control cultures were transfected with pSV2hyg only. Transfected cells were allowed to grow for 1 week in complete growth medium before drug selection (50 $\mu$g ml$^{-1}$ hygromycin B). After 3–4 weeks individual colonies were isolated by ring cloning and expanded.

*Molecular characterisation of* neu-*T-expressing cell lines*

Ten micrograms of genomic DNA was prepared from *neu*-T-expressing cell lines using the Nucleon kit and digested with *Eco*RI and *Xba*I. Southern blotting was performed on the digested DNA (Sambrook *et al.*, 1989) and the blots were hybridised with a $^{32}$P-labelled, random primed, c-*neu* cDNA probe (labelled with the 'Prime-it' kit), and hybridised in 'Quik-hyb' solution. Total RNA was prepared from cell lines using guanidinium thiocyanate (Chomczynski and Sacchi, 1987). Northern blotting was carried out on 10 $\mu$g of this RNA (Sambrook *et al.*, 1989) and blots were hybridised to a radiolabelled c-*neu* probe.

*Immunochemicals, immunoblotting and immunoprecipitation*

Immunoblotting for c-*neu* used the mouse monoclonal antibody, c-*neu* Ab-3, which recognises both the rat protein and c-*erb*B-2. Immunoblotting for anti-phosphotyrosine was achieved with the recombinant anti-phosphotyrosine peroxidase-conjugated antibody, RC20. The mouse monoclonal antibody c-*neu* Ab-4, raised to the external domain of the rat c-*neu* gene product, was used for immunoprecipitation studies. Indirect immunofluorescence detected binding of c-*neu* Ab-4 using a goat anti-mouse IgG (whole molecule) biotin-conjugated antibody followed by streptavidin fluorescein protein. For FACS analysis c-*neu* Ab-4 was visualised with a goat anti-mouse IgG (Fc specific) FITC-conjugated antibody on freshly trypsinised live cells after staining on ice.

For immunoprecipitation, $1-2 \times 10^6$ cells were washed with phosphate-buffered saline (PBS) containing 1 mM sodium orthovanadate and lysed in lysis buffer (10 mM disodium hydrogen phosphate, 10 mM sodium dihydrogen phosphate, 150 mM sodium chloride, 1% (v/v) Nonidet P-40, 10% (v/v) glycerol, 50 mM sodium fluoride, 10 mM sodium pyrophosphate, protease inhibitors (40 $\mu$M leupeptin, 10 $\mu$g ml$^{-1}$ aprotinin and 1 mM phenylmethylsulphonyl fluoride) and tyrosine phosphatase inhibitors (1 mM sodium orthovanadate and 10 mM benzamidine hydrochloride)). Approximately 250 $\mu$g of total protein lysate was mixed, by rotation, with 1–2 $\mu$g of antibody, at 4°C for 1 h. Antibody-bound protein was captured by rotation with 50 $\mu$l of protein A-Sepharose CL-4B beads (10 mg ml$^{-1}$ in lysis buffer), at 4°C, for 2–3 h. Beads were pelleted by centrifugation at 11 000 r.p.m. for 5 min at 4°C, and washed five times in lysis buffer before being prepared for SDS–PAGE (Harlow and Lane, 1988). After SDS– PAGE, proteins were transferred to Immobilon-P transfer membrane by conventional wet blotting technique (Harlow and Lane, 1988). Detection of *neu*-T and phosphotyrosine was performed using the ECL chemiluminescence system according to manufacturer's instructions.

*Growth assays*

Replicates of $1 \times 10^5$ cells were allowed to attach overnight to 25 cm$^2$ culture flasks in RPMI-1640 containing 10% (v/v) fetal calf serum (basal medium). The following day the cells were washed with PBS and then re-fed with RPMI-1640 medium, containing various additives. For single-point growth measurements, cells were set up in quadruplicate, harvested after 7 days and counted using a haemocytometer. For multipoint growth curves, cells were set up in triplicate, harvested at daily intervals, and counted as above. For multi-well growth experiments cells were set up at 2 or $5 \times 10^4$ cells per well in 24-well culture plates, in triplicate, overnight in basal medium. The following day cells were washed with PBS, and re-fed with RPMI-1640 containing additives specified. Results were obtained by staining cells with methylene blue [0.5% (w/v) in 50% ethanol] for 2 h, followed by rinsing in water to remove excess stain. Staining was quantified spectrophotometrically by solubilising with lauryl sarkosyl [1% (v/v) in PBS] and measuring the absorbance at 620 nm.

*Assays of tumorigenicity*

Cell lines were assayed for the acquisition of anchorage-independent growth using Noble agar [0.3% (w/v) in complete medium] as described by Freshney (1983). They were assayed for tumorigenicity *in vivo* by inoculating them subcutaneously into both nude and severe combined immunodeficient (SCID) mice at $1 \times 10^6$ cells per site as described by Topley *et al.* (1993) either with or without basement membrane extract (Matrigel) as a vehicle.

*Analysis of apoptosis*

Cells which had detached into the growth medium were harvested by centrifugation at 1800 r.p.m. for 5 min at room temperature. The resulting cell pellets were prepared as for electron microscopy by fixation with 2.5% (v/v) glutaraldehyde in PBS. After fixation cell pellets were treated for 1 h in 1% (w/v) osmium tetroxide, dehydrated through a series of graded ethanols and embedded in Epon–Araldite as described by Ormerod *et al.* (1994). Sections of 1 $\mu$m were then cut, dried onto a glass slide and stained with 1% (w/v) toluidine blue in 1% (v/v) borax before visualisation by light microscopy. To determine DNA degradation, cells were harvested from both the supernatant growth medium and cell monolayer, and genomic DNA prepared (Catchpoole and Stewart, 1993). The internucleosomal fragmentation of DNA obtained was assessed by the presence of laddering using the $^{32}$P end-labelling method described by Rosi *et al.* (1992). Flow cytometry was used to record DNA histograms produced by cells fixed with 70% ethanol (v/v) and stained with propidium iodide (Ormerod *et al.*, 1992).

### Results

*Isolation of variant cell lines*

The transforming potential of *neu*-T expression was assessed by transfection of pSV2*neu*-T, together with pSV2hyg, into the non-transformed immortalised human mammary luminal epithelial cell line, HB4a. After a period of drug selection lasting 2 weeks, drug-resistant clones were picked at random and screened by indirect immunofluorescence. *neu*-T expression was detected in two out of four clones screened in this manner. These two *neu*-T-expressing clones, N4.1 and N4.2, were grown on and established as variant cell lines. Both the cell lines exhibited morphological characteristics typical of transformed cells, notably the loss of the regular epithelial organisation seen in the hygromycin B-resistant control cell line (H4.1) established by a similar protocol from cells transfected with pSV2hyg only. The alteration of cellular morphology was most marked in the N4.1 cell line (Figure 1).

**Figure 1** Changes in cellular morphology induced by expression of *neu*-T. Phase-contrast micrographs of the H4.1 (**a**) and N4.1 (**b**) cell lines derived from the non-transformed immortalised human mammary luminal epithelial cell line, HB4a. Bar = 100 μm.

*Expression of* neu-*T in HB4a and variant cell lines*

Southern and Northern transfer hybridisations confirmed that the N4.1 and N4.2 cell lines had integrated the *neu*-T sequence (approximately 5–10 copies) into their chromosomal DNA and expressed *neu*-T mRNA (data not shown). Immunoprecipitation experiments showed that the *neu*-T protein expressed in these cell lines was phosphorylated on tyrosine residues, indicating that, as expected, the protein is constitutively kinase active (Figure 2). FACS analysis of live cells showed expression of *neu*-T in the N4.1 and N4.2 cell lines at the cell surface. Using the same method, the H4.1 cell line gave a mean FACS channel value approximately equal to that seen with a control sample, where the c-*neu*-specific first antibody (NFA) had been omitted, confirming that this human cell line does not react with the antibody against rodent c-*neu*, while both *neu*-T-expressing variant cell lines showed elevated values. The mean FACS channel values were: NFA = 34, H4.1 = 37, N4.1 = 191, N4.2 = 131 on an arithmetic (non-log) scale. Owing to its higher levels of *neu*-T expression, N4.1 was selected for further detailed characterisation.

*Effect of* neu-*T expression on cell growth and tumorigenicity*

The growth characteristics of the N4.1 cell line were initially studied using single-point growth experiments over a fixed time period of 7 days (Figure 3). These experiments showed that in complete growth medium the N4.1 cell line grew faster and reached much higher cell densities than the H4.1 cell line, indicating a loss of contact inhibition of growth. Similar experiments performed in basal medium (i.e. without hydrocortisone, insulin and cholera toxin) revealed that the N4.1 cell line continued to proliferate to confluence, while the H4.1 cell line (like the parental HB4a cell line) arrested before this point, indicating that the N4.1 cell line had acquired reduced growth factor dependency. Proliferation rates were examined by multipoint growth curves from which the doubling times in the exponential growth phase were calculated. In complete growth medium, the N4.1 cell line was found to have a much shorter doubling time (36 h) than the H4.1 cell line (68 h), the latter being very similar to the parental HB4a cell line. Further examination of the reduction in growth factor dependency of the N4.1 cell line showed that, while the addition of hydrocortisone and insulin to growth medium acted synergistically in the promotion of proliferation and overgrowth, the presence of cholera toxin reduced both the proliferative rate and the final cell density.

**Figure 2** Expression and activation of *neu*-T in HB4a-derived cell lines. c-*neu* was immunoprecipitated from HB4a-derived cell lines as described. Immunocomplexes were run on SDS–PAGE, Western blotted and probed for either c-*neu* [lanes 1–4 (anti-c-neu)] or phosphotyrosine [lanes 5–8 (anti-P-Tyr)].

**Figure 3** Alterations in growth characteristics induced by expression of *neu*-T. Bar charts show the results of single-point cell number determinations performed in quadruplicate (error bars represent mean ± s.d.) on the H4.1 and N4.1 cell lines after 7 days in culture. Cells were cultured in RPMI-1640 with 10% (v/v) fetal calf serum (▨, basal), containing either hydrocortisone and insulin alone (□, HC & I), or with cholera toxin in addition (■, complete).

In contrast, cholera toxin stimulated the proliferation of the H4.1 control cell line, again like the parental stocks of HB4a (Figure 3). These changes in growth characteristics were also observed, but to a lesser extent, in the N4.2 cell line, which expressed lower levels of *neu*-T on the cell membrane (see above).

The N4.1 cell line displayed altered growth properties *in vitro* compared with the control cell line which had undergone a similar process of selection, isolation and expansion, which indicated that *neu*-T expression had resulted in transformation of the cells. This was tested further by analysing anchorage-independent growth and growth as xenografts. While the H4.1 cell line gave colonies in soft agar at only very low efficiency (0.15% ± 0.08 s.d.), the N4.1 cell line produced colonies with a diameter greater than 0.5 mm at almost 100-fold higher efficiency (12.16% ± 1.81 s.d.). However, when assayed for tumorigenic transformation *in vivo*, by subcutaneous injection into either nude or SCID mice, neither the H4.1 cell line nor the N4.1 cell line produced tumours in 20 animals inoculated (15 nudes and five SCIDs).

*Induction of apoptosis by serum withdrawal in cells transformed by* neu-*T expression and its abrogation by glucocorticoids*

Since, when cultured in serum-containing medium, the N4.1 cell line clearly showed reduced requirements for exogenous growth factors, the effects of reduced serum concentrations were examined. In low-serum medium (LSM; RPMI-1640 medium containing 0.2% (v/v) fetal calf serum) the H4.1 cell line entered growth arrest (this was proved to be quiescence since addition of complete medium to H4.1 cells arrested with LSM caused them to recommence growth: data not shown), while the N4.1 cell line underwent extensive cell death. This showed all the hallmarks of apoptosis: loss of cell–cell contact, membrane blebbing, cytoplasmic condensation, and eventually cell fragmentation, resulting in the presence of a large number of small, membrane-bounded apoptotic bodies in the supernatant medium (Figure 4a and b). These supernatants were collected, and examination of the fixed cells by high-resolution light microscopy revealed that the chromatin they contained had become marginalised and condensed (Figure 4d). Apoptosis of the N4.1 cell line was evident after 3 days in LSM. By contrast, the supernatant fraction obtained from the H4.1 cell line under the same conditions was much smaller and contained only cell debris which showed no obvious signs of apoptosis (Figure 4c).

To examine the time course of apoptosis, the effects of serum withdrawal on the N4.1 cell line were investigated further by measuring the condensation of the chromatin associated with apoptosis. Samples were taken, at daily intervals, from cultures of the H4.1 and N4.1 cell lines that had been maintained in LSM, and the DNA content of these cells was analysed by flow cytometry after staining with propidium iodide (Figure 5). On day 1 of the experiment, both the H4.1 and the N4.1 cell lines produced DNA histograms containing the expected $G_1$, S-phase and $G_2/M$ populations. By day 5, the H4.1 cell line showed a reduction in both S-phase (from 33% to 0% of the total cell population) and $G_2$ (from 26% to 10%) populations, indicating $G_1$ arrest. The N4.1 cell line continued to show an S-phase fraction (albeit reduced from 17% to 4% of the total cell population) with a reduced $G_2$ population (from 33% to 5%) and the appearance of a sub-$G_1$ peak (14%), which has been observed in other examples of apoptosis (Ormerod *et al.*, 1992).

Another parameter associated with apoptotic cell death is the internucleosomal cleavage of DNA. The H4.1 and the N4.1 cell lines were, therefore, compared using a sensitive radiolabelling method. Cell lines were cultured in LSM and again sampled at daily intervals. Substantial internucleosomal fragmentation was evident in the N4.1 cells sampled from day 3 onwards, while in contrast, even after 5 days, little or

**Figure 5** Effects of serum withdrawal on cell cycle progression. DNA histograms are shown from cells of the H4.1 and N4.1 cell lines cultured in RPMI-1640 containing 0.2% (v/v) fetal calf serum for 1, 3 and 5 days. DNA content of cells was measured by staining DNA with propidium iodide (PI) and using flow cytometry to quantitate the PI-DNA fluorescence.

**Figure 4** Morphological changes during apoptotic cell death induced by serum withdrawal. Phase-contrast micrographs of H4.1 (**a**) and N4.1 (**b**) cell lines (bar = 100 μm) after 3 days in RPMI-1640 containing 0.2% (v/v) fetal calf serum. **c** and **d** show light micrographs of glutaraldehyde-fixed non-adherent cells from corresponding cultures (bar = 20 μm).

no such specific cleavage of DNA was seen in the H4.1 cell line (Figure 6).

To ascertain whether exogenous growth factors were capable of preventing apoptosis, a series of multiwell experiments were performed, in which the adherent cells and hence the amount of proliferation or apoptosis was assessed by fixing and staining cells with methylene blue. This protocol was used to study the effects of a wide range of peptide growth factors and hormones added to LSM. These included insulin, epidermal growth factor (EGF), nerve growth factor (NGF), acidic fibroblast growth factor (a-FGF), basic fibroblast growth factor (b-FGF), transforming growth factor alpha (TGF-$\alpha$) and transforming growth factor beta (TGF-$\beta$) (all tested in replicate at a range of concentrations between 100 and 0.01 $\mu g\ ml^{-1}$) as well as hydrocortisone, dexamethasone, corticosterone, aldosterone, deoxycorticosterone, oestrone, oestradiol, progesterone, testosterone, androstenedione and dehydroepiandrosterone (all initially tested at a concentration of 3 $\mu$M). Only hydrocortisone and other steroids with glucocorticoid activity (e.g. dexamethasone, corticosterone and, to a lesser extent, aldosterone) were found to inhibit the induction of apoptosis and promote proliferation of the N4.1 cell line. The oestrogens, androgens and their precursors were all inactive, as was progesterone. A dose–response study using hydrocortisone showed that this anti-apoptotic effect could be produced by concentrations equivalent to those seen physiologically (20–100 ng $ml^{-1}$) (Figure 7).

**Figure 6** Induction of internucleosomal fragmentation of DNA by serum withdrawal. Electrophoresis of $^{32}P$ end-labelled DNA obtained from both adherent and non-adherent cells of the H4.1 (lane 1) and N4.1 (lane 7) cell lines cultured in RPMI-1640 containing either 10% (v/v) fetal calf serum (lanes 1 and 7) or 0.2% (v/v) fetal calf serum (H4.1, lanes 2–6 and N4.1, lanes 8–12), representing days 0–5 of serum deprivation. The figure shows a negative of the autoradiograph produced.

**Figure 7** Inhibition of the induction of apoptosis by hydrocortisone. Hydrocortisone concentration curves are shown for the H4.1 (●) and N4.1 (○) cell lines after 7 days culture in RPMI-1640 containing 0.2% (v/v) fetal calf serum supplemented with varying concentrations of hydrocortisone. Cell survival and proliferation were determined by staining cells with methylene blue and measuring the absorbence at 620 nm in triplicate wells (error bars represent mean ± s.d.).

### Discussion

The initial aim of this present study was to define further the role of c-*erb*B-2 in the progression of malignancy in human breast tumours. *neu*-T, the mutated, constitutively active rat homologue of c-*erb*B-2, was expressed in the conditionally immortalised human mammary luminal epithelial cell line, HB4a, to enable comparisons to be made with other forms of the receptor. Expression of *neu*-T was found to cause alterations in cellular morphology and growth characteristics in variant cell lines found to express detectable surface $p185^{neu\text{-}T}$. In one such variant, N4.1, these changes included an increase in proliferative rate, an increase in saturation density, a reduction in growth factor requirements, the acquisition of anchorage-independent growth and an altered response to cholera toxin as seen in other transformed cells (Pastan and Willingham, 1978). However, neither the N4.1 cell line nor a variant of the HB4a cell line transformed with point mutated c-Ha-*ras* (R4.2) (data not shown) produced tumours in nude mice. This failure to acquire a fully malignant phenotype *in vivo* has also been observed in studies with these oncogenes using the spontaneously immortalised, non-transformed human mammary epithelial cell line, MCF-10A (Ciardellio *et al*., 1992; Normanno *et al*., 1994). This is in contrast to with the findings of D'souza *et al*. (1993), and shows that the role of c-*erb*B-2 overexpression (or mutationally activated *neu*-T) in the acquisition of tumorigenicity in human mammary epithelial cells is as yet unresolved. However, an unexpected observation was that the *neu*-T-expressing cell lines underwent a marked apoptotic response when serum levels were reduced to 0.2% (v/v) in another transformation assay.

Apoptosis, or programmed cell death, is a mode of physiological cell death characterised by the condensation of chromatin around the nuclear periphery and the cleavage of DNA into oligonucleosome-size fragments by an endogenous endonuclease. During apoptosis, cells exhibit extensive blebbing of the plasma membrane accompanied by nuclear and cytoplasmic budding to produce dense, membrane-bounded apoptotic bodies (Kerr *et al*., 1972; Arends and Wyllie, 1991). Many of the genetic alterations commonly associated with malignancy have been shown to affect apoptosis. For example, deregulated expression of the c-*myc* protooncogene accelerates apoptosis in cells that are deprived of serum or growth factors (Evan *et al*., 1992), while expression of the gene *bcl*-2 or mutated forms of the tumour suppressor p53 can inhibit apoptosis (Wagner *et al*., 1993; Yonish-Rouach *et al*., 1993). Apoptosis also occurs naturally during involution of the lactating rodent mammary gland following weaning (Walker *et al*., 1989) and in the human breast epithelium during the menstrual cycle (Anderson *et al*., 1982). However, no specific mechanisms that induce or inhibit apoptosis in the normal mammary gland have yet been identified, although studies with the MCF-7 cell line have indicated that oestrogen withdrawal may promote apoptosis in xenografts of some stocks of this cell line (Kyprianou *et al*., 1991). Apoptotic cell death was not observed *in vitro* with this cell line. In the present study the N4.1 cell line was found to undergo rapid and extensive apoptotic cell death when deprived of serum in culture. Although the induction of apoptosis by serum withdrawal has been described for fibroblast cell lines transformed by proto-oncogene expression (Evan *et al*., 1992), a link between receptor protein tyrosine kinases and apoptosis in epithelial cells has not previously been reported.

The N4.1 cell line has an increased growth rate in serum-containing medium when compared with a control cell line

(H4.1). This is consistent with the model that expression of *neu*-T promotes entry into the cell cycle by constitutively activating mitogenic signalling pathways. The removal of serum growth factors signals growth arrest, and it is conceivable that it is the reception of these two incompatible growth-regulatory signals that causes the N4.1 cell line to undergo apoptosis upon serum withdrawal. This 'conflict hypothesis' was put forward by Evan *et al.* (1992) to explain a similar response seen with Rat-1 fibroblasts transformed by c-*myc*. Recently, the 'conflict hypothesis' for the induction of apoptotic cell death has been taken further (Evan and Littlewood, 1994) to produce the 'dual hypothesis', which implies that a protein may have dual function, performing either a proliferative or apoptotic role depending on the cellular environment. Thus, in the present study, the expression of *neu*-T produces proliferation in the presence of serum, while in the absence of serum it results in apoptosis. These studies indicate that the expression of *neu*-T can have a dual function, and provides additional evidence that, although as yet ill-defined, the signalling pathways for cellular proliferation, differentiation and death are linked (Yonish-Rouach *et al.*, 1993). Since *neu*-T is known to act upstream of c-Ha-*ras* in the mitogenic signal transduction pathway (Ben-Levy *et al.*, 1994), the observation that a variant of the HB4a cell line R4.2 transformed by point mutated c-Ha-*ras* does not appear to undergo apoptosis in low-serum medium (data not shown) suggests that the apoptotic signalling pathway in this system is not dependent on activation of c-Ha-*ras*.

The capacity to undergo apoptosis may have contributed to the failure of the N4.1 cell line to produce tumours in nude mice, and indicates that, to progress to a fully malignant phenotype *in vivo*, additional genetic alterations are necessary. One mutational event commonly found in a wide range of human malignancies, including breast cancer, is the inactivation of the tumour suppressor p53 (Hollstein *et al.*, 1991). p53 has been implicated in apoptotic cell death (Yonish-Rouach *et al.*, 1991). However, since p53 function is known to be impaired by the presence of the SV40 large T antigen (Segawa *et al.*, 1993), which is present and active in the HB4a cell line under the conditions used in this study, it is likely that the serum withdrawal-induced apoptosis observed in the N4.1 cell line occurs in a p53-independent manner.

Previous studies, using fibroblast and haemopoietic cell lines, have shown that growth factors such as insulin-like growth factor I (IGF-I) (Harrington *et al.*, 1994) and colony-stimulating factors (CSFs) (Williams *et al.*, 1990), respectively, are effective in inhibiting apoptosis. However, in the current study, the ability to prevent apoptosis induced by serum withdrawal in the epithelially derived N4.1 cell line was found only with glucocorticoids. Glucocorticoids have been shown to increase the stability of c-*erb*B-2 mRNA transcripts and strongly stimulate the expression of the epidermal growth factor receptor (c-*erb*B-1) (Ewing *et al.*, 1989; Karlan *et al.*, 1994). It is possible that, by stabilising and increasing the expression of *erb*B family members, glucocorticoids enhance the mitogenic signals produced, even in conditions in which ligands are limiting, such as those experienced during serum withdrawal. Added EGF, however, did not by itself abrogate apoptosis in this system. This glucocorticoid abrogation of epithelial apoptotic cell death stands in marked contrast to its role in inducing thymocyte programmed cell death (Wyllie, 1980) but is in accordance with the finding that glucocorticoids inhibit the apoptotic cell death involved in the involution of the mouse mammary gland (Ossowski *et al.*, 1979).

Although expression of *neu*-T does not directly mimic the overexpression of c-*erb*B-2 observed in many breast tumours, the N4.1 cell line described above does provide a reproducible model linking receptor protein tyrosine kinases to *in vitro* transformation and the induction of apoptosis in human mammary luminal epithelial cells. A fuller understanding of the signalling pathways involved in apoptotic cell death in such cells might enable future drugs to be designed specifically to activate these pathways since many current therapeutic agents have been shown to cause cell death by apoptosis (Hickman, 1992).

**Acknowledgements**
We would like to thank David Linstead for his help with morphological observations, Jenny Titley for the running of flow cytometry, David Robertson for light microscopy and Thomas Eichholtz for his advice during preparation of the manuscript. We are also grateful to Barry Gusterson and Ken Powell for their support and encouragement.

### References

ANDERSON TJ, FERGUSON DJ AND RAAB GM. (1982). Cell turnover in the resting human breast: influence of parity, contraceptive pill, age and laterality. *Br. J. Cancer*, **46**, 376–382.

ARENDS MJ AND WYLLIE AH. (1991). Apoptosis: mechanisms and roles in pathology. *Int. Rev. Exp. Pathol.*, **32**, 223–254.

BEN-LEVY R, PATERSON HF, MARSHALL CJ AND YARDEN Y. (1994). A single autophosphorylation site confers oncogenicity to the Neu/ErbB-2 receptor and enables coupling to the MAP kinase pathway. *EMBO J.*, **13**, 3302–3311.

BOUCHARD L, LAMARRE L, TREMBLAY PJ AND JOLICOEUR P. (1989). Stochastic appearance of mammary tumors in transgenic mice carrying the MMTV/*c-neu* oncogene. *Cell*, **57**, 931–936.

CATCHPOOLE DR AND STEWART BW. (1993). Etoposide-induced cytotoxicity in two human T-cell leukemic lines: delayed loss of membrane permeability rather than DNA fragmentation as an indicator of programmed cell death. *Cancer Res.*, **53**, 4287–4296.

CHOMCZYNSKI P AND SACCHI N. (1987). Single-step method of RNA isolation by acid guanidinium thiocyanate–phenol–chloroform extraction. *Anal. Biochem.*, **162**, 156–159.

CIARDELLO F, GOTTARDIS M, BASOLO F, PEPE S, NORMANNO N, DICKSON RB, BIANCO AR AND SALOMON DS. (1992). Additive effects of c-*erbB*-2, c-Ha-*ras*, and transforming growth factor-α on in vitro transformation of human mammary epithelial cells. *Mol. Carcinogen.*, **6**, 43–52.

D'SOUZA B, BERDICHEVSKY F, KYPRIANOU N AND TAYLOR-PAPADIMITRIOU J. (1993). Collagen-induced morphogenesis and expression of the $\alpha_2$-integrin subunit is inhibited in c-erbB-2-transfected human mammary epithelial cells. *Oncogene*, **8**, 1797–1806.

DIFIORE PP, PIERCE JH, KRAUS MH, SEGATTO O, KING CR AND AARONSON SA. (1987). c-*erbB-2* is a potent oncogene when overexpressed in NIH/3T3 cells. *Science*, **237**, 178–182.

EVAN GI AND LITTLEWOOD TD. (1993). The role of c-*myc* in cell growth. *Curr. Opin. Genet. Dev.*, **3**, 44–49.

EVAN GI, WYLLIE AH, GILBERT CS, LITTLEWOOD TD, LAND H, BROOKS M, WATERS CM, PENN LZ AND HANCOCK DC. (1992). Induction of apoptosis in fibroblasts by c-*myc* protein. *Cell*, **69**, 119–128.

EWING TM, MURPHY LJ, NG ML, PANG GY, LEE CS, WATTS CK AND SUTHERLAND RL. (1989). Regulation of epidermal growth factor receptor by progestins and glucocorticoids in human breast cancer cell lines. *Int. J. Cancer*, **44**, 744–752.

FRESHNEY RI. (1983). *Culture of Animal Cells. A Manual of Basic Technique*. Alan R. Liss: New York.

GULLICK WJ, LOVE SB, WRIGHT C, BARNES DM, GUSTERSON B, HARRIS AL AND ALTMAN DG. (1991). *c-erbB-2* protein overexpression in breast cancer is a risk factor in patients with involved and uninvolved lymph nodes. *Br. J. Cancer*, **63**, 434–438.

GUSTERSON BA, GELBER RD, GOLDHIRSCH A, PRICE KN, SAEVE-SOEDERBORGH J, ANABAZHAGAN R, STYLES J, RUDENSTAM CM, GOLOUH R, REED R, MARTINEZ-TELLO F, TILTMAN A, TORHORST J, GRIGOLATO P, BETTELHEIM R, NEVILLE AM, BURKI K, CASTIGLIONE M, COLLINS J, LINDTNER J AND SENN HJ. (1992). Prognostic importance of *c-erbB-2* expression in breast cancer. *J. Clin. Oncol.*, **10**, 1049–1056.

HARLOW E AND LANE D. (1988). *Antibodies: A Laboratory Manual*. Cold Spring Harbor Laboratory Press: Cold Spring Harbor, NY.

HARRINGTON EA, BENNET MR, FANIDI A AND EVAN GI. (1994). c-Myc-induced apoptosis in fibroblasts is inhibited by specific cytokines. *EMBO J.*, **13**, 3286–3295.

HICKMAN JA. (1992). Apoptosis induced by anticancer drugs. *Cancer Metastasis Rev.*, **11**, 121–139.

HOLLSTEIN M, SIDRANSKY D, VOGELSTEIN B AND HARRIS CC. (1991). p53 mutations in human cancers. *Science*, **253**, 49–53.

HUDZIAK RM, SCHLESSINGER J AND ULLRICH A. (1987). Increased expression of the putative growth factor receptor $p185^{HER2}$ causes transformation and tumorigenesis of NIH 3T3 cells. *Proc. Natl. Acad. Sci. USA*, **84**, 7159–7163.

KARLAN BY, JONES J, SLAMON DJ AND LAGASSE LD. (1994). Glucocorticoids stabilise HER-2/neu messenger RNA in human epithelial ovarian carcinoma cells. *Gynecol. Oncol.*, **53**, 70–77.

KERR JFR, WYLLIE AH AND CURRIE AR. (1972). Apoptosis: a basic biological phenomenon with wide-ranging implications in tissue kinetics. *Br. J. Cancer*, **26**, 239–257.

KYPRIANOU N, ENGLISH HF, DAVIDSON NE AND ISAACS JT. (1991). Programmed cell death during regression of the MCF-7 human breast cancer following estrogen ablation. *Cancer Res.*, **51**, 162–166.

MULLER WJ, SINN E, PATTENGALE PK, WALLACE AND LEDER P. (1988). Single-step induction of mammary adenocarcinoma in transgenic mice bearing the activated *c-neu* oncogene. *Cell*, **54**, 105–115.

NORMANNO N, SELVAM MP, QI C, SAEKI T, JOHNSON G, KIM N, CIARDIELLO F, SHOYAB M, PLOWMAN G, BRANDT R, TORARDO G AND SALOMON DS. (1994). Amphiregulin as an autocrine growth factor for c-Ha-*ras*- and c-*erb*B-2-transformed human mammary epithelial cells. *Proc. Natl Acad. Sci. USA*, **91**, 2790–2794.

O'HARE MJ, ORMEROD MG, MONAGHAN P, LANE EB AND GUSTERSON BA. (1991). Characterization *in vitro* of luminal and myoepithelial cells isolated from the human mammary gland by cell sorting. *Differentiation*, **46**, 209–221.

ORMEROD MG, COLLINS MLK, RODRIGUEZ-TARDUCHY G AND ROBERTSON D. (1992). Apoptosis in interleukin-3-dependent haemopoietic cells. Quantification by two flow cytometric methods. *J. Immunol. Methods*, **153**, 57–65.

ORMEROD MG, O'NEILL CF, ROBERTSON D AND HARRAP KR. (1994). Cisplatin induces apoptosis in a human ovarian carcinoma cell line without concomitant internucleosomal degradation of DNA. *Exp. Cell Res.*, **211**, 231–237.

OSSOWSKI L, BIEGEL D AND REICH E. (1979). Mammary plasminogen activator: correlation with involution, hormonal modulation and comparison between normal and neoplastic tissue. *Cell*, **16**, 929–940.

PASTAN I AND WILLINGHAM M. (1978). Cellular transformation and the 'morphologic phenotype' of transformed cells. *Nature*, **274**, 645–650.

PIERCE JH, ARNSTEIN P, DIMARCO E, ARTRIP J, KRAUS MH, LONARDO F, DIFIORE PP AND AARONSON SA. (1991). Oncogenic potential of erbB-2 in human mammary epithelial cells. *Oncogene*, **6**, 1189–1194.

ROSL F. (1992). A simple and rapid method for the detection of apoptosis in human cells. *Nucleic Acids Res.*, **20**, 5243.

SAMBROOK J, FRITSCH EF AND MANIATIS T. (1989). *Molecular Cloning: A Laboratory Manual*, 2nd edn. Cold Spring Harbor Laboratory Press: Cold Spring Harbor, NY.

SCHECHTER AL, STERN DF, VAIDYANATHAN L, DECKER SJ, DREBIN JA, GREENE MI AND WEINBERG RA. (1984). The *Neu* oncogene: an erbB-related gene encoding a 185,000- Mr tumour antigen. *Nature*, **312**, 513–516.

SEGAWA K, MINOWA A, SUGASAWA K, TAKANO T. AND HANAOKA F. (1993). Abrogation of p53-mediated transactivation by SV40 large T antigen. *Oncogene*, **8**, 543–548.

STAMPS AC, DAVIES SC, BURMAN J AND O'HARE MJ. (1994). Analysis of proviral integration in human mammary epithelial cell lines immortalized by retroviral infection with a temperature-sensitive SV40T-antigen construct. *Int. J. Cancer*, **57**, 865–874.

TOPLEY P, JENKINS DC, JESSUP EA AND STABLES JN. (1993). Effect of reconstituted basement membrane components on the growth of a panel of human tumour cell lines in nude mice. *Br. J. Cancer*, **67**, 953–958.

ULLRICH A AND SCHLESSINGER J. (1990). Signal transduction by receptors with tyrosine kinase activity. *Cell*, **61**, 203–212.

WAGNER AJ, SMALL MB AND HAY N. (1993). Myc-mediated apoptosis is blocked by ectopic expression of bcl-2. *Mol. Cell. Biol.*, **13**, 2432–2440.

WALKER NI, BENNETT RE AND KERR JFR. (1989). Cell death by apoptosis during involution of the lactating breast in mice and rats. *Am. J. Anat.*, **185**, 19–32.

WILLIAMS GT, SMITH CA, SPOONCER E, DEXTER TM AND TAYLOR D. (1990). Haemopoietic colony stimulating factors promote cell survival by suppressing apoptosis. *Nature*, **343**, 76–79.

WYLLIE AH. (1980). Glucocorticoid-induced thymocyte apoptosis is associated with endogenous endonuclease activation. *Nature*, **284**, 555–556.

YAMAMOTO T, IKAWA S, AKIYAMA T, SEMBA K, NOMURA N, MIYAJIMA N, SAITO T AND TOYOSHIMA K. (1986). Similarity of protein encoded by the human *c-erbB-2* gene to epidermal growth factor receptor. *Nature*, **319**, 230–234.

YONISH-ROUACH E, RESNITZKY D, LOTEM J, SACHS L, KIMCHI A AND OREN M. (1991). Wild-type p53 induces apoptosis of myeloid leukaemic cells that is inhibited by interleukin-6. *Nature*, **352**, 345–347.

YONISH-ROUACH E, GRUNWALD D, WILDER S, KIMCHI A, MAY E, LAWRENCE J, MAY P AND OREN M. (1993). p53-mediated cell death: relationship to cell cycle control. *Mol. Cell. Biol.*, **13**, 1415–1423.